# Impact of Abnormal Remote Stress Myocardial Blood Flow by Dynamic CT Perfusion on Clinical Outcomes

**DOI:** 10.1038/s41598-020-66992-w

**Published:** 2020-06-24

**Authors:** Nobuo Tomizawa, Shengpu Chou, Yusuke Fujino, Satoshi Matsuoka, Kodai Yamamoto, Shinichi Inoh, Takeshi Nojo, Kanako K. Kumamaru, Shinichiro Fujimoto, Sunao Nakamura

**Affiliations:** 1 Department of Radiology, New Tokyo Hospital, Chiba, Japan; 2 Department of Radiology, Juntendo University Graduate School of Medicine, Tokyo, Japan; 3 Department of Diabetes, New Tokyo Hospital, Chiba, Japan; 4 Department of Cardiology, Kashiwa Kousei General Hospital, Chiba, Japan; 5 Department of Cardiology, New Tokyo Hospital, Chiba, Japan; 6 Department of Cardiovascular Medicine, Juntendo University Graduate School of Medicine, Tokyo, Japan

**Keywords:** Ischaemia, Outcomes research

## Abstract

The objective of this study was to investigate the incremental prognostic value for adverse events of myocardial blood flow (MBF) derived from stress computed tomography perfusion (CTP) at remote myocardium over cardiac risk factors and ischemia. We prospectively analyzed 242 patients who underwent dynamic CTP and CT angiography. Adverse events were defined as a composite of all-cause mortality, non-fatal myocardial infarction, unstable angina, heart failure requiring hospitalization, peripheral artery disease, and stroke. MBF value was calculated in each myocardial segment and ischemia was defined as mild decrease in MBF in two consecutive segments or moderate decrease in a single segment accompanied with a coronary stenosis ≥50%. The mean MBF of the non-ischemic segments was defined as remote MBF. We divided the patients into two groups by median MBF value of 1.15 ml/min/g. During a median follow-up of 18 months, 18 patients had adverse events. Annual event rate showed a significant difference between patients with low (≤1.15 ml/min/g) and high (>1.15 ml/min/g) MBF (6.1% vs 1.8%, *p* = 0.02). Univariate analysis showed that low MBF was a significant predictor of events (hazard ratio (HR): 3.4; 95% confidence interval (CI): 1.2 to 12.0; *p* = 0.02). This relationship maintained significant after adjusted for the presence of ischemia and cardiac risk factors (HR: 3.0; 95%CI: 1.1 to 11.1; *p* = 0.04). In conclusion, MBF value ≤1.15 ml/min/g derived from dynamic CTP in remote myocardium is significantly related with poor outcome and this relationship was independent of myocardial ischemia and cardiac risk factors.

## Introduction

Myocardial computed tomography perfusion (CTP) is an emerging technique to assess ischemia-causing stenosis using CT^1–6^. Although the sensitivity to diagnose ischemia-causing stenosis of coronary CT angiography is very high, the low specificity ranging from 60 to 70% is a major shortcoming^1–3^. Recent meta-analyses showed that myocardial CTP could improve the specificity to 80% without significantly reducing the sensitivity^1–3^.

The evaluation of CTP can be achieved either by static or dynamic acquisitions^4^. Static CTP evaluates relative ischemia by determining hypoperfusion at first-pass of contrast medium. Dynamic CTP could further evaluate the absolute myocardial blood flow (MBF)^4,6^. Quantitative evaluation of myocardial ischemia could be performed using MBF of stenosis-related myocardium. Conversely, microvascular function of the myocardium could be evaluated using MBF of the remote myocardium, which is myocardium without coronary stenosis. Previous studies using positron emission tomography (PET) showed that reduced coronary flow reserve (CFR) was associated with increased risk of cardiovascular events^7,8^. Recent studies using CTP showed that ischemia detected by CTP was related to incremental predictive value in assessing future events over clinical risk factors and coronary stenosis^9,10^. However, the prognostic value of MBF at the remote myocardium is not fully assessed. Therefore, the purpose of the present study was to investigate the incremental prognostic value for adverse events of MBF at the remote myocardium over cardiac risk factors and ischemia.

## Methods

### Patients

**Figure 1:**
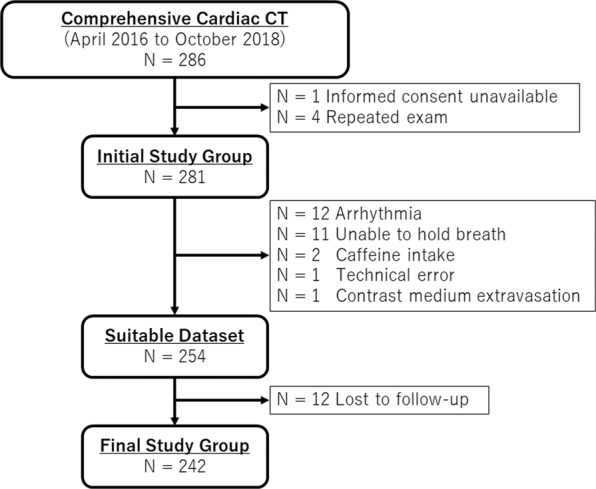
A total of 44 patients were excluded from the initial cohort. The final study group included 242 patients. CT, computed tomography.

A total of 286 patients between April 2016 and October 2018 undergoing comprehensive cardiac CT (a combination of dynamic myocardial CTP and coronary CT angiography) entered in a prospective registry under the Protocol Registration System of the University hospital Medical Information Network Clinical Trials Registry (UMIN000024245). The inclusion criteria were history of type 2 diabetes, regardless of symptoms, suspected coronary artery disease due to multiple risk factors, or evaluation of coronary stenosis after percutaneous coronary intervention and/or coronary artery bypass grafting. Patients who met the inclusion criteria without severe renal dysfunction (estimated glomerular filtration rate >40 ml/min/1.73m^2^) were invited at the outpatient department (Fig. 1). The study protocol was approved by the New Tokyo Hospital Ethics Committee and all patients gave written informed consent. The study protocol conforms to the ethical guidelines of the 1975 Declaration of Helsinki. All patients were asked to discontinue caffeine intake at least 12 hours before the exam.

### CT acquisition

All patients underwent cardiac CT using a single-source CT (Somatom Definition AS + ; Siemens Healthineers, Forchheim, Germany) with a collimation of 64×0.6 mm and flying-focal spot, resulting in 128 slices^6^. All scans were performed at the fastest gantry rotation time of 300 ms. Two intravenous lines were placed in both antecubital veins for administration of contrast medium and adenosine. We used a self-monitoring device for the patient to visually control the respiration (Abches; APEX Medical, Tokyo, Japan). We trained each patient before the scan to keep the end-inspiratory position at the same position. We used iopamidol 370 mg iodine/ml (Iopamiron 370; Bayer, Osaka, Japan) when the body weight was <70 kg, otherwise iomeprol 350 mg iodine/ml (Iomeron 350; Eisai, Tokyo, Japan). We used iomeprol in obese patients because the amount of contrast medium included was 135 ml for iomeprol but was 100 ml for iopamidol. We adjusted the amount of contrast medium by body weight to maintain the signal-to-noise ratio in obese patients.

Myocardial CTP started 3 min after administration of adenosine triphosphate (Adetphos; Kowa Company, Tokyo, Japan) at 140 μg/kg/min. When the heart rate increase was <10 beats/min or blood pressure decrease <10 mmHg, we increased the injection rate of adenosine up to 200 μg/kg/min. The scanning parameters were as follows: tube voltage, 100 kVp; reference mAs, 190 mAs; scan coverage 68.5 mm; acquisition window, 30‒40% of the R-R interval. A total of 38‒60 ml of contrast medium was injected for 12 s, followed by a saline chaser. The scan initiation timing after the contrast medium injection was optimized with the stress heart rate: 6, 8, and 10 s when >90, 65-90, and <65 beats/min, respectively. During the data acquisition of 25 s, 5 sets of 2-slab prospective electrocardiogram-gated axial scans were performed. The time interval between each slab was ≈1 s, and the interval was ≈3 s between each set. Adenosine triphosphate infusion was discontinued after the acquisition was completed. Half-reconstruction images were acquired with a slice thickness of 0.75 mm, and an increment of 0.4 mm using a cardiac kernel (I36f) with sinogram-affirmed iterative reconstruction (SAFIRE) strength 2.

Coronary CT angiography was performed by prospective electrocardiogram-gated axial scan. When the tube current-time product of the CTP scan exceeded 150 mAs, the tube potential and the reference mAs was set as 120 kVp and 250 mAs, otherwise these were set as 100 kVp and 350 mAs. The acquisition window initiated at 60‒75% of the R-R interval when the heart rate was below 65 beats/min, otherwise initiated at 30‒75%. A total of 37‒85 ml of contrast medium was injected for 14 s, followed by a saline flush. Bolus tracking method was performed to determine the scan timing. The scan started 6 s after the descending aorta reached 60 Hounsfield units (HU) above the initial value. If the heart rate was over 65 beats/min, a maximum dose of 12.5 mg of landiolol (Corebeta; Ono Pharmaceutical, Tokyo, Japan) was given intravenously^11^. No side-effects were reported using beta-blockers including vasospasm. All patients received 0.3 mg sublingual nitroglycerin (Nitropen; Nippon Kayaku, Tokyo, Japan). Half-reconstruction was performed with a slice thickness of 0.75 mm and an increment of 0.4 mm using a cardiac kernel (I36f) with SAFIRE strength 2.

For processing, images were transferred to a workstation (Synapse VINCENT Ver 5.2; Fujifilm Medical, Tokyo, Japan). The effective radiation dose was derived by multiplying the dose-length product with the constant k (k = 0.014).

### CT stenosis analysis

Two experienced cardiovascular interpreters evaluated the presence of significant stenosis (≥50% stenosis) on a per-vessel basis. When the evaluation differed between the two readers, the final stenosis grade was determined by consensus.

### CTP analysis

**Figure 2:**
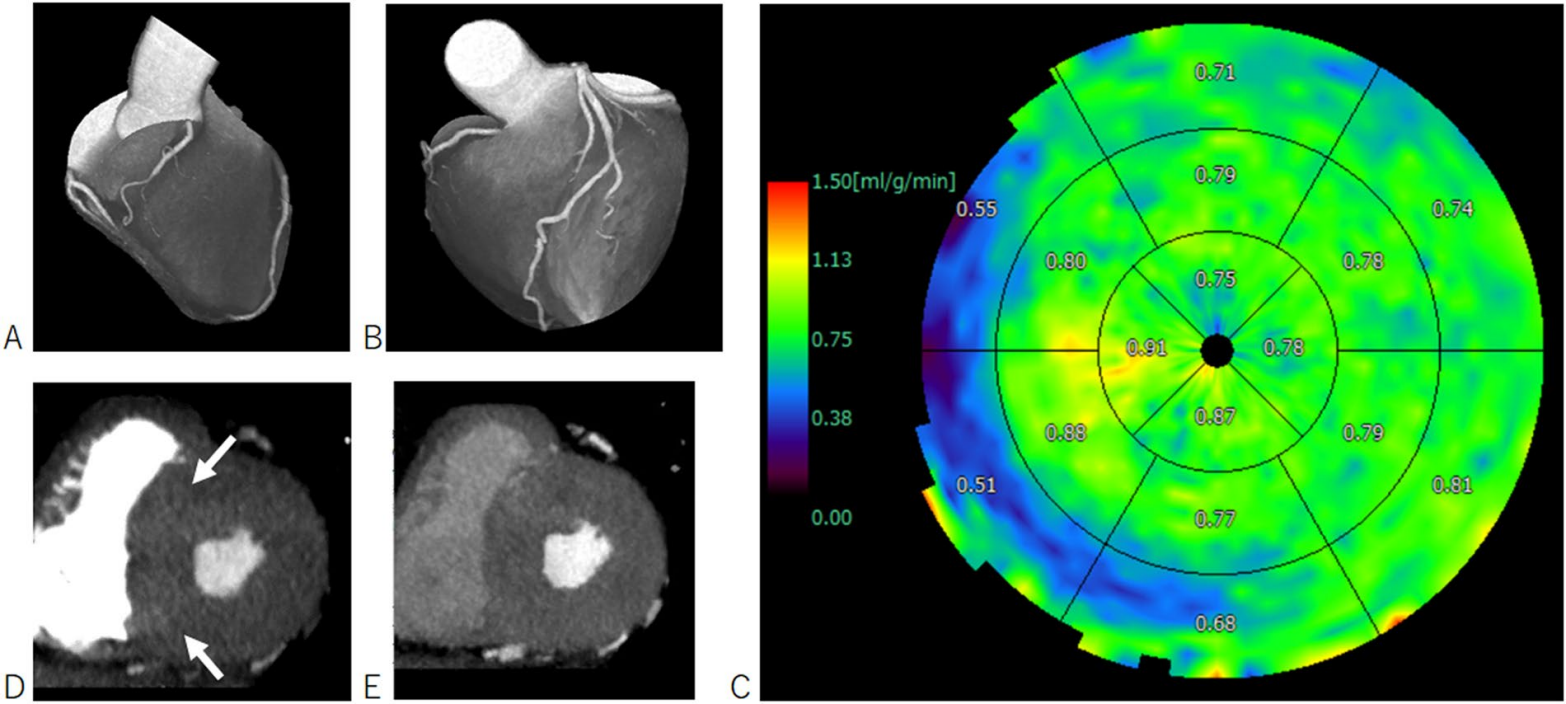
76-year old male diagnosed diabetes mellitus during hospitalization of Fournier’s gangrene. Surface maximal intensity projection images show no coronary stenosis (A,B). Bull’s eye view shows diffuse decrease of stress myocardial blood flow (C). We determined the abnormal low blood flow at the basal septum as an artifact, because streak artifact from the dense contrast medium of the right ventricle (D, arrows) was present. No perfusion defect is noted at the later phase (E). The myocardial blood flow of this patient was calculated as 0.80 ml/min/g. The patient suffered sudden cardiac death 752 days after the exam.

A dedicated parametric deconvolution technique was used to calculate the absolute MBF using a software (Perfusion analysis; Fujifilm Medical). To increase the precision of the fit, double sampling of the arterial input function was performed^6^. The input function was sampled in the left ventricle at every table position and combined into one arterial input function that had twice the sampling rate of the tissue time-attenuation-curve. Regional MBF was measured using the standard American College of Cardiology/American Heart Association 17-segment model^12^. The software automatically calculated the MBF of each myocardial segment (Fig. 2). A coronary stenosis ≥50% accompanied with 2 consecutive segments with a MBF decrease of 20% or 1 segment with a MBF decrease of 35% compared to the remaining segments were determined ischemic^13^. We calculated the mean value of the non-ischemic segments to obtain the MBF of the remote myocardium. We carefully excluded segments with abnormally low MBF due to streak artifact from the dense contrast medium of the right ventricle (Fig. 2).

### Follow-up and endpoints

Follow-up information was obtained by clinical visits at 3-month intervals up to March 2019. All reported events were checked by hospital records or, if possible, by direct contacts with the primary physician. The primary endpoint was a composite of all-cause mortality, non-fatal myocardial infarction, unstable angina, heart failure requiring hospitalization, peripheral artery disease, and stroke. All revascularization procedures were ischemia driven. We did not determine early revascularization within 3 months after CT as an event and did not exclude these patients for analysis. The diagnosis of non-fatal myocardial infarction was based on the presence of typical chest pain, elevated cardiac enzymes, and typical electrocardiogram changes^14^. Unstable angina was defined as new-onset, worsening, or angina at rest that required hospital admission. We also analyzed all-cause mortality as hard events.

### Statistical analysis

Continuous variables were shown as means ± SD and categorical variables as number unless otherwise described. Student’s *t* test was used to compare continuous variables. The chi-square test or Fisher’s exact test was used to compare categorical variables. We divided the patients into two groups according to MBF value: low MBF group as MBF ≤ 1.15 ml/min/g and high MBF group as MBF > 1.15 ml/min/g. We chose this value because 1.15 ml/min/g was the median MBF in this cohort.

Kaplan-Meier survival analysis was used to assess the distribution of time to events in different groups. Differences in time-to-event curves were compared with the log-rank statistic. The effect of the variables on the primary endpoint was evaluated using the univariate Cox proportional hazards models. Adjusted Cox proportional hazards models were used to evaluate whether the predictive value of MBF was independent of the presence of ischemia and cardiac risk factors: Model 1, MBF ≤ 1.15 and presence of ischemia; Model 2, parameters in model 1 and sex, age >65 y, cardiac risk factors (diabetes mellitus, hypertension, dyslipidemia, smoking, and family history); Model 3, parameters in model 2 and known cardiovascular disease, performance of early revascularization.

Sample size was calculated based on the results of the FACTOR-64 trial^15^. We assumed that the mean event rate would be around 2%, and the event rate of the high and low MBF group would be 4% and 0.5%, respectively. To indicate a difference between the different MBF groups, the minimum sample size was calculated as 498 patients (249 patients in each group) at 0.80 power. Sample size calculations were based on a type 2 error (α = 0.05). However, patient recruitment became impossible because of the change in the affiliation of the primary investigator in May 2019. We therefore included 286 patients for analysis.

Statistical analyses were performed using JMP software (ver. 12.2.0; SAS, Cary, NC). In all analysis, *p* < 0.05 was adopted to indicate statistical significance.

## Results

### Patient population

Records of 286 patients in the registry were initially examined (Fig. 1). We excluded patients who refused to participate in the study (n = 1) and underwent a repeated CTP exam (n = 4). Furthermore, the following 27 patients were excluded: arrhythmia (n = 12), unable to hold breath (n = 11), caffeine intake <12 h before the exam (n = 2), technical error (n = 1), and contrast medium extravasation (n = 1). An additional 12 patients were lost to follow-up. The final study population consisted of 242 patients.

### Patient and scan characteristics

Male patients accounted for 70% and the mean age was 65.9 ± 11.3 y (Table 1). Symptomatic patients consisted 22% of the population. Most patients had diabetes mellitus (84%), hypertension (77%), and dyslipidemia (81%). A history of previous cardiovascular disease was found in 35 patients (14%). Body mass index was lower in patients with adverse events than patients without (*p* = 0.02), otherwise, no statistically significant difference was observed in the remaining patient characteristics between the groups.

**Table 1:** Patient Characteristics.

	All patients N = 242	Adverse event N = 18	No adverse event N = 224	p
Male	170 (70)	16 (89)	154 (69)	0.11
Age (y)	65.9 ± 11.3	70.5 ± 9.1	65.5 ± 11.4	0.07
Body mass index (kg/m^2^)	25.4 ± 4.7	22.9 ± 3.9	25.6 ± 4.7	0.02*
Symptom				
Atypical	28 (12)	0 (0)	1 (0.5)	0.12
Typical	1 (0.4)	1 (6)	27 (12)	
Palpitation	11 (5)	1 (6)	10 (4)	0.58
Dyspnea	14 (6)	1 (6)	13 (6)	1.0
Cardiac risk factors				
Diabetes mellitus	203 (84)	17 (94)	186 (84)	0.33
Hypertension	186 (77)	14 (78)	172 (77)	0.92
Dyslipidemia	195 (81)	12 (67)	183 (82)	0.13
Smoking, current/ex	63 (26)/91 (38)	3 (17)/7 (39)	60 (27)/84 (38)	0.58
Family history	59 (24)	4 (22)	55 (25)	1.0
Known CVD	35 (14)	3 (17)	32 (14)	0.73
Early revascularization	27 (11)	16 (89)	199 (89)	1.0
Remote MBF (ml/min/g)	1.17 ± 0.33	1.10 ± 0.30	1.18 ± 0.33	0.31
Ischemia at CTP	41 (17)	4 (22)	37 (17)	0.52
CTA ≥ 50% stenosis				
All	97 (40)	9 (50)	88 (39)	0.46
Single	49 (20)	5 (28)	44 (20)	0.64
Multiple	48 (20)	4 (22)	44 (20)	

Numbers are reported as mean ± SD or N (%).

*Statistically significant, p < 0.05.

CTA, computed tomography angiography; CTP, computed tomography perfusion; CVD, cardiovascular disease; MBF, myocardial blood flow.

The mean contrast medium injected during CTP and CT angiography was 51.8 ± 4.0 and 51.9 ± 8.7 ml, respectively, with a total of 103.7 ± 12.2 ml (Table 2). The mean effective dose of CTP and CT angiography was 2.7 ± 0.8 and 3.3 ± 1.5 mSv, respectively, with a total of 7.6 ± 2.3 mSv. The systolic blood pressure dropped 15 mmHg and the heart rate increased 10 beats/min during stress.

**Table 2:** Scan Characteristics.

Contrast medium (ml)	
CTP	51.8 ± 4.0
CTA	51.9 ± 8.7
Total	103.7 ± 12.2
Effective dose (mSv)	
CTP	2.7 ± 0.8
CTA	3.3 ± 1.5
Total	7.6 ± 2.3
ATP (μg/min/kg)	
140	212 (88)
160	21 (9)
180	8 (3)
200	1 (0.4)
Blood pressure (mmHg)	
Rest, systolic	131 ± 22
Rest, diastolic	71 ± 12
Stress, systolic	116 ± 21
Stress, diastolic	61 ± 12
Heart rate (beats/min)	
Rest	68.4 ± 11.7
Stress	77.8 ± 12.6
CTA	62.4 ± 23.3

Numbers are reported as mean ± SD or N (%).

ATP, adenosine triphosphate; CTA, computed tomography angiography; CTP, computed tomography perfusion.

### CTP and CT angiography results

The mean value of MBF at remote myocardium was 1.17 ± 0.33 ml/min/g (Table 1). CT angiography showed significant (≥50%) stenosis in 97 patients (40%). Of these stenoses, 41 patients (41%) were proven to have ischemia on stress CTP. These values were not statistically significant between patients with and without adverse events.

### Outcomes

The median follow-up was 18 months (interquartile range, 12–24 months). Twenty-seven patients (11%) underwent early intervention, which we did not determine as an event. A total of 18 patients had adverse events: cardiac death (n = 1), non-cardiac death (n = 1), non-fatal myocardial infarction (n = 1), unstable angina (n = 6), heart failure (n = 2), peripheral artery disease (n = 3), and stroke (n = 4). One patient with stroke died due to complications from a stroke.

### Cardiac CT findings and adverse events

**Figure 3:**
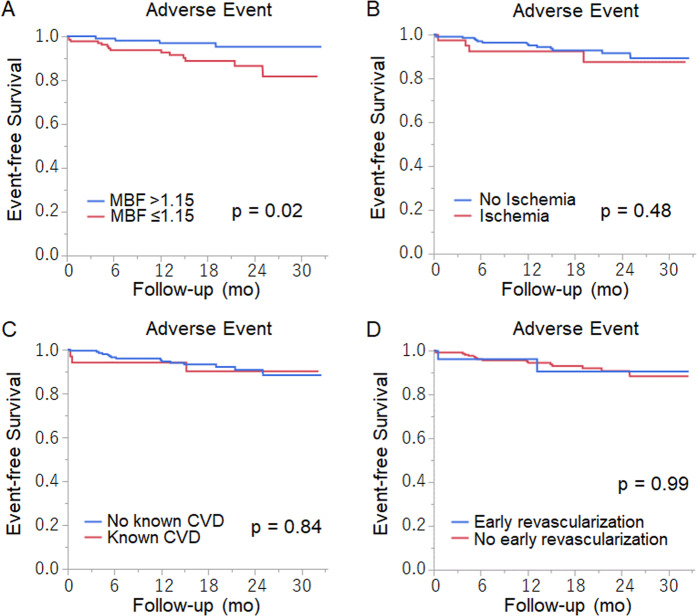
Kaplan-Meier curves for adverse events according to MBF (A), presence of ischemia (B), known previous cardiovascular disease (C), and early revascularization (D). MBF, myocardial blood flow.

Kaplan-Meier curves by MBF (Fig. 3A) showed that annualized event rate in patients with low MBF (≤1.15 ml/min/g) was significantly higher than patients with high MBF (>1.15 ml/min/g) (6.1% vs 1.8%, *p* = 0.02). The event rates did not differ between patients with or without ischemia, known cardiovascular disease, and early revascularization (Fig. 3B–D).

Univariate analysis showed that low MBF (≤1.15 ml/min/g) (hazard ratio (HR): 3.4; 95% confidence interval (CI): 1.2 to 12.0; *p* = 0.02) was a significant predictor of adverse events (Table 3). The other variables including the presence of ischemia, sex, age, cardiac risk factors, known cardiovascular disease, and early revascularization were not significant predictors of adverse events.

**Table 3:** Univariate Predictors of Adverse Events.

	HR (95%CI)	p
MBF ≤1.15	3.4 (1.2‒12.0)	0.02*
Ischemia	1.5 (0.4‒4.2)	0.50
Male	3.4 (1.0‒21.6)	0.06
Age >65 y	2.2 (0.8‒7.8)	0.14
Cardiac risk factors		
Diabetes mellitus	3.2 (0.6‒57.3)	0.18
Hypertension	0.9 (0.3‒3.4)	0.93
Dyslipidemia	0.5 (0.2‒1.3)	0.15
Smoking	0.8 (0.3‒2.0)	0.55
Family history	0.9 (0.2‒2.4)	0.80
Known CVD	1.1 (0.3‒3.5)	0.84
Early revascularization	1.0 (0.16‒3.5)	1.0

*Statistically significant, p < 0.05.

CI, confidence interval; CVD, cardiovascular disease; HR, hazard ratio; MBF, myocardial blood flow.

Multivariate analyses were performed to evaluate whether MBF was an independent predictor of adverse events (Table 4). When adjusted for the presence of ischemia (Model 1), low MBF (≤1.15 ml/min/g) remained a significant predictor of adverse events (HR; 3.4, 95%CI: 1.2 to 11.9; *p* = 0.02). Even when adjusted for sex, age, and cardiac risk factors (Model 2), low MBF remained a significant predictor of adverse events (HR: 3.0; 95%CI: 1.1 to 11.1; *p* = 0.04). Furthermore, when adjusted for known cardiovascular disease and early revascularization (Model 3), low MBF remained an independent predictor of adverse events (HR: 3.0; 95%CI: 1.1 to 11.0; *p* = 0.04), and male was also an independent predictor (HR: 4.5; 95%CI: 1.1 to 31.7; *p* = 0.04).

**Table 4:** Multivariate Predictors of Adverse Events.

	Model 1		Model 2		Model 3	
	HR (95%CI)	p	HR (95%CI)	p	HR (95%CI)	p
MBF ≤1.15	3.4 (1.2‒11.9)	0.02*	3.0 (1.1‒11.1)	0.04*	3.0 (1.1‒11.0)	0.04*
Ischemia	1.4 (0.4‒4.0)	0.55	1.5 (0.4‒4.4)	0.50	1.9 (0.5‒6.3)	0.33
Male			4.1 (1.0‒28.0)	0.05	4.5 (1.1‒31.7)	0.04*
Age >65 y			2.0 (0.7‒7.2)	0.21	2.1 (0.7‒7.4)	0.19
Cardiac risk factors						
Diabetes mellitus			4.1 (0.8‒75.0)	0.09	4.3 (0.4‒78.0)	0.09
Hypertension			1.0 (0.3‒3.8)	0.96	1.1 (0.4‒4.3)	0.83
Dyslipidemia			0.5 (0.2‒1.6)	0.23	0.5 (0.2‒1.6)	0.24
Smoking			0.5 (0.2‒1.3)	0.14	0.4 (0.2‒1.3)	0.12
Family history			1.0 (0.3‒2.7)	0.94	1.1 (0.3‒3.1)	0.93
Known CVD					0.8 (0.2‒2.7)	0.80
Early revascularization					0.5 (0.1‒2.2)	0.40

*Statistically significant, p < 0.05.

CI, confidence interval; CVD, cardiovascular disease; HR, hazard ratio; MBF, myocardial blood flow.

### MBF value and all-cause mortality

**Figure 4:**
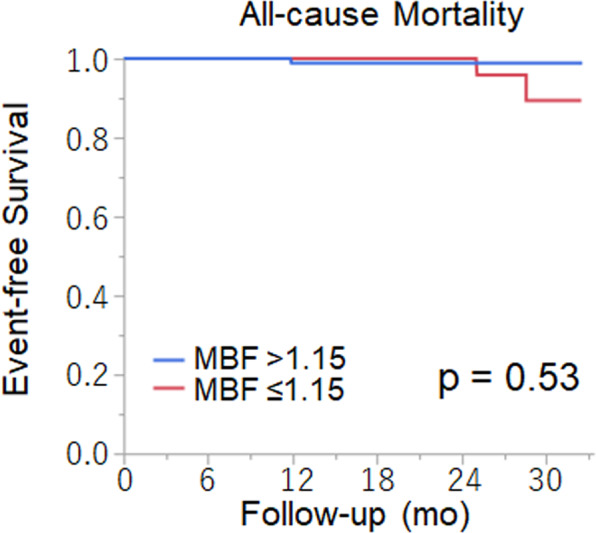
Kaplan-Meier curve for all-cause mortality according to MBF. MBF, myocardial blood flow.

In the low MBF group, one patient died due to sudden cardiac attack (Fig. 2), and the other patient died due to stroke-related complications. In the high MBF group, one patient died due to malignant lymphoma. Kaplan-Meier curves by MBF showed that the annualized event rate for all-cause mortality in patients with low MBF was higher than patients with high MBF, but the difference did not reach significance (4.0% vs 0.4%, *p* = 0.53) (Fig. 4).

## Discussion

The present study showed that decreased MBF of the remote myocardium was a significant predictor of adverse events independent of myocardial ischemia and conventional cardiac risk factors. The annualized event rate for adverse events increased from 1.8% to 6.1% in patients with a MBF value of ≤1.15 ml/min/g than the remaining patients. Most patients in the present study had diabetes mellitus without symptoms and the results of FACTOR-64 trial were against to screen these patients by coronary CT angiography^15^. The annual event rate was higher in this study than the FACTOR-64 trial, because we included patients with known previous cardiovascular disease. The present study emphasized the importance of evaluating microvascular function and managing microvascular disease might improve the prognosis of patients with diabetes mellitus.

Presence of ischemia by dynamic CTP was not related with increased future events in the present study. This might be in conflict with a previous study which showed that patients with at least one perfusion defect at dynamic CTP were at increased risk of major adverse cardiovascular events (HR: 2.50; 95%CI: 1.34 to 1.64)^16^. This relationship remained significant after adjusting for age, sex, and cardiac risk factors. In the study by Meinel *et al*.^16^ more than half of the events were percutaneous coronary intervention (21 out of 40 events), and all coronary revascularizations including early and late procedures were determined as events. Therefore, the results of the CTP exam could have resulted in increased coronary interventions. Another study showed that the presence of abnormal perfusion was related to an increased risk of major adverse cardiovascular events independent of significant coronary stenosis at CT angiography (HR: 5.7; 95%CI: 1.9 to 16.9)^9^. In this study by Nakamura *et al*.^9^ early coronary interventions (within 60 days after CT) were not determined as events, but follow-up was censored at the time of intervention. We did not censor patients with early coronary interventions in the analysis and the annual event rate did not differ between patients with or without early coronary interventions. Our result is in line with the ISCEMIA trial^17^ which showed that cardiovascular risk of an initial invasive strategy as compared with an initial conservative strategy did not differ in patients with intermediate stenosis and proven ischemia.

Microvascular function is affected by various factors. Cardiac risk factors such as hypertension and diabetes mellitus are risk factors for microvascular dysfunction^18,19^. Longer diabetes duration is related to MBF reduction at stress^20^. Furthermore, coronary intervention procedure might decrease the regional MBF^21^. Several studies showed that glycemic improvement normalizes microvascular function in diabetic patients^22,23^. Medications such as statins^24^ or glucagon-like peptide-1 agonists^25^ might improve microvascular function. Nicorandil moderately improved exercise-induced myocardial ischemia in microvascular angina patients by dilating both epicardial and resistance vessels and modulating the vasomotor responses of the vessels to sympathetic stimulation^26^. Currently, a randomized controlled trial is ongoing to investigate the role of phosphodiesterase-5 inhibitor to reduce myocardial ischemia in female microvascular angina patients^27^. Improvement of microvascular function by these methods might be related to better prognosis but this is only hypothetical and needs further investigation.

One major shortcoming of dynamic CTP is the increase in radiation dose than static CTP protocols. A meta-analysis showed that the mean radiation dose ranged from 5.3 to 10.5 mSv per dynamic CTP exam^2^. Recently, dynamic CTP protocols with lower sampling rate have been introduced^28,29^. The mean radiation dose could be reduced to 2 to 3 mSv using these protocols. However, low sampling rate might reduce the MBF value^30^ and furthermore, MBF values might differ between various calculation methods^31^. Further study is necessary to normalize the value of MBF derived from dynamic CTP.

This study had a number of limitations. First, this was a single-center study and we used only one CT scanner for the evaluation of stress MBF. Further multicenter studies using different CT scanners and various MBF calculation methods are required to validate the results of this study. Second, the difference in mortality rate did not reach significance between patients with low and high MBF. Studies with a larger cohort and longer follow-up period might solve this problem. Third, patients who underwent early revascularization were included for analysis in this study. Although no adverse events related to coronary stents were reported during follow-up, the results might change when the follow-up period is longer. Fourth, most patients in this cohort had diabetes mellitus. Validation is necessary to confirm the results of this study in patients without diabetes mellitus. Finally, we chose 1.15 ml/min/g as a cutoff for impaired microvascular function because it was a median value in the present cohort. Further study is necessary to determine the absolute value for impaired microvascular function.

In conclusion, an MBF value ≤1.15 ml/min/g derived from dynamic CTP in remote myocardium is significantly related with poor outcome. The relationship was independent of myocardial ischemia and cardiac risk factors. This emphasizes the importance of evaluating the microvascular function in patients especially with diabetes mellitus.

## References

[1] Sørgaard MH (2016). Diagnostic accuracy of static CT perfusion for the detection of myocardial ischemia. A systematic review and meta-analysis. J. Cardiovasc. Comput. Tomogr.

[2] Lu M, Wang S, Sirajuddin A, Arai AE, Zhao S (2018). Dynamic stress computed tomography myocardial perfusion for detecting myocardial ischemia: A systematic review and meta-analysis. Int. J. Cardiol..

[3] Gonzalez JA (2015). Meta-Analysis of Diagnostic Performance of Coronary Computed Tomography Angiography, Computed Tomography Perfusion, and Computed Tomography-Fractional Flow Reserve in Functional Myocardial Ischemia Assessment Versus Invasive Fractional Flow Reserve. Am. J. Cardiol..

[4] Danad I, Szymonifka J, Schulman-Marcus J, Min JK (2016). Static and dynamic assessment of myocardial perfusion by computed tomography. Eur. Hear. J. – Cardiovasc. Imaging.

[5] Pontone G (2019). Incremental Diagnostic Value of Stress Computed Tomography Myocardial Perfusion With Whole-Heart Coverage CT Scanner in Intermediate- to High-Risk Symptomatic Patients Suspected of Coronary Artery Disease. JACC Cardiovasc. Imaging.

[6] Tomizawa, N. *et al*. Feasibility of dynamic myocardial CT perfusion using single-source 64-row CT. *J. Cardiovasc. Comput. Tomogr*. in press 10.1016/j.jcct.2018.10.003 30309765

[7] Herzog BA (2009). Long-Term Prognostic Value of 13N-Ammonia Myocardial Perfusion Positron Emission Tomography. J. Am. Coll. Cardiol..

[8] Bajaj NS (2018). Coronary Microvascular Dysfunction and Cardiovascular Risk in Obese Patients. J. Am. Coll. Cardiol..

[9] Nakamura S (2019). Incremental Prognostic Value of Myocardial Blood Flow Quantified With Stress Dynamic Computed Tomography Perfusion Imaging. JACC Cardiovasc. Imaging.

[10] Meinel FG (2017). Global quantification of left ventricular myocardial perfusion at dynamic CT imaging: Prognostic value. J. Cardiovasc. Comput. Tomogr.

[11] Tomizawa, N., Hayakawa, Y., Inoh, S., Nojo, T. & Nakamura, S. Clinical utility of landiolol for use in coronary CT angiography. *Res. Reports Clin. Cardiol*. 145, 10.2147/RRCC.S77559 (2015).

[12] MACHAC J (2006). Positron emission tomography myocardial perfusion and glucose metabolism imaging. J. Nucl. Cardiol.

[13] Segawa C (2009). [Basic investigation of software named “Heart Risk View” to estimate the probability of cardiac events, for the purpose of evaluating the availability in clinical practice]. Kaku Igaku..

[14] Antman E (2000). Myocardial infarction redefined—a consensus document of The Joint European Society of Cardiology/American College of Cardiology committee for the redefinition of myocardial infarction. J. Am. Coll. Cardiol..

[15] Muhlestein JB (2014). Effect of Screening for Coronary Artery Disease Using CT Angiography on Mortality and Cardiac Events in High-Risk Patients With Diabetes. JAMA.

[16] Meinel FG (2017). Prognostic Value of Stress Dynamic Myocardial Perfusion CT in a Multicenter Population With Known or Suspected Coronary Artery Disease. Am. J. Roentgenol..

[17] Maron DJ (2020). Initial Invasive or Conservative Strategy for Stable Coronary Disease. N. Engl. J. Med..

[18] Vliegenthart R (2016). Dynamic CT myocardial perfusion imaging identifies early perfusion abnormalities in diabetes and hypertension: Insights from a multicenter registry. J. Cardiovasc. Comput. Tomogr.

[19] Assante R (2017). Coronary atherosclerotic burden vs. coronary vascular function in diabetic and nondiabetic patients with normal myocardial perfusion: a propensity score analysis. Eur. J. Nucl. Med. Mol. Imaging.

[20] Tomizawa N (2018). Longer diabetes duration reduces myocardial blood flow in remote myocardium assessed by dynamic myocardial CT perfusion. J. Diabetes Complications.

[21] Li Y (2019). Prevalence of Decreased Myocardial Blood Flow in Symptomatic Patients with Patent Coronary Stents: Insights from Low-Dose Dynamic CT Myocardial Perfusion Imaging. Korean J. Radiol..

[22] Morais NV (2012). Glycemic improvement normalizes myocardial microvascular reserve in type 2 diabetes. Int. J. Cardiol..

[23] Schindler TH (2007). Improvement in coronary vascular dysfunction produced with euglycaemic control in patients with type 2 diabetes. Heart.

[24] Lario FC (2013). Atorvastatin treatment improves myocardial and peripheral blood flow in familial hypercholesterolemia subjects without evidence of coronary atherosclerosis. Echocardiography.

[25] Gejl M (2012). Exenatide alters myocardial glucose transport and uptake depending on insulin resistance and increases myocardial blood flow in patients with type 2 diabetes. J. Clin. Endocrinol. Metab..

[26] Jaw-Wen C (1997). Effects of short-term treatment of nicorandil on exercise-induced myocardial ischemia and abnormal cardiac autonomic activity in microvascular angina. Am. J. Cardiol..

[27] Park S-J (2014). Understanding of chest pain in microvascular disease proved by cardiac magnetic resonance image (UMPIRE): study protocol for a randomized controlled trial. Trials.

[28] Tomizawa N (2019). Feasibility of dynamic myocardial CT perfusion using single-source 64-row CT. J. Cardiovasc. Comput. Tomogr.

[29] Hubbard L, Malkasian S, Zhao Y, Abbona P, Molloi S (2019). Timing optimization of low-dose first-pass analysis dynamic CT myocardial perfusion measurement: validation in a swine model. Eur. Radiol. Exp.

[30] Yokoi T (2019). Impact of the sampling rate of dynamic myocardial computed tomography perfusion on the quantitative assessment of myocardial blood flow. Clin. Imaging.

[31] Eck BL (2018). The role of acquisition and quantification methods in myocardial blood flow estimability for myocardial perfusion imaging CT. Phys. Med. Biol..

